# IMPACT OF LEUKOCYTE INFLAMMATORY MARKERS ON OUTCOMES OF CRITICALLY ILL PATIENTS WITH GASTROINTESTINAL DISEASES

**DOI:** 10.1590/S0004-2803.24612026-051

**Published:** 2026-08-24

**Authors:** Anna Clara Rios ROCHA, Liana CODES, Fernanda SALES, Cláudio Celestino ZOLLINGER, Paulo Lisboa BITTENCOURT

**Affiliations:** 1 Escola Bahiana de Medicina e Saúde Pública, Salvador, BA, Brasil.; 2 Hospital Português da Bahia, Salvador, BA, Brasil.

**Keywords:** Leucocyte ratios, prognosis, outcome, critical care, organ failure, mortality, Razões leucocitárias, prognóstico, desfecho, cuidados intensivos, falência orgânica, mortalidade

## Abstract

**Background:**

Leukocyte markers, such as neutrophil-to-lymphocyte ratio (NLR), monocyte-to-lymphocyte ratio (MLR), and systemic immune inflammation index (SII), are related to systemic inflammation and may predict organ failure (OF) and mortality in critically ill patients. This study aims to evaluate the association of NLR, MLR, and SII with OF and mortality in medical and surgical patients admitted to a specialty ICU dedicated to the treatment of gastrointestinal (GI) disorders.

**Methods:**

All patients admitted to a specialty GI ICU in Brazil, from January 2016 to December 2023, were retrospectively evaluated. Baseline white blood cell counts, NLR, MLR, and SII were calculated in all subjects and compared to different outcomes, including OF and in-hospital mortality.

**Results:**

6,703 patients (3,593 women, mean age 67.4 ± 18.3 years) were included in the study. NLR, MLR, and SII were significantly higher in subjects with sepsis and circulatory failure (CF). Likewise, higher NLR and SII were also observed in patients with kidney (KF) and respiratory (RF) failure. NLR, MLR and SII were associated with in-hospital mortality, but only NLR was independently associated with CF (OR: 1.017; 95%CI: 1.013-1.021; *P*<0.0001), RF (OR: 1.007; 95%CI: 1.001-1.012; *P*<0.0001) and with an increased risk of death (OR: 1.016; 95%CI: 1.012-1.019; *P*<0.0001) in multivariate analysis.

**Conclusion:**

Leukocyte markers, particularly NLR and MLR, were associated with OF and mortality in critically ill patients with gastrointestinal disorders, but only NLR was independently associated with CF, RF, and mortality.

## HIGHLIGHTS

• NLR, MLR, and SII were associated with organ failure and sepsis in GI ICU patients.

• Baseline NLR independently predicted circulatory failure, respiratory failure, and mortality.

• Leukocyte-derived inflammatory markers provide inexpensive prognostic information at ICU admission.

## RESUMO

**Contexto:**

Marcadores leucocitários, como a relação neutrófilo-linfócito (RNL), a relação monócito-linfócito (RML) e o índice sistêmico de inflamação imune (ISI), estão associados à inflamação sistêmica e podem predizer falência orgânica (FO) e mortalidade em pacientes críticos. Este estudo teve como objetivo avaliar a associação entre RNL, RML e ISI com FO e mortalidade em pacientes internados em uma UTI especializada em doenças gastrointestinais (GI), tanto clínicos quanto cirúrgicos.

**Métodos:**

Todos os pacientes internados em uma UTI especializada em GI no Brasil, no período de janeiro de 2016 a dezembro de 2023, foram avaliados retrospectivamente. As contagens basais de leucócitos, RNL, RML e ISI foram calculadas em todos os participantes e comparadas a diferentes desfechos, incluindo FO e mortalidade hospitalar.

**Resultados:**

6.703 pacientes (3.593 mulheres, com idade média de 67,4 ± 18,3 anos) foram incluídos no estudo. Os índices NLR, MLR e SII foram significativamente maiores em indivíduos com sepse e insuficiência circulatória (IC). Da mesma forma, níveis mais elevados de NLR e SII também foram observados em pacientes com insuficiência renal (IR) e insuficiência respiratória (IRp). NLR, MLR e SII estiveram associados à mortalidade hospitalar, mas apenas o NLR apresentou associação independente com IC (OR: 1,017; IC95%: 1,013-1,021; *P*<0,0001), IRp (OR: 1,007; IC95%: 1,001-1,012; *P*<0,0001) e com risco aumentado de óbito (OR: 1,016; IC95%: 1,012-1,019; *P*<0,0001) na análise multivariada.

**Conclusão:**

Os marcadores leucocitários RNL e RML estiveram associados à FO e à mortalidade em pacientes críticos com distúrbios gastrointestinais, mas apenas a RNL apresentou associação independente com FO, IRp e mortalidade.

## INTRODUCTION

Several leukocyte indices, including neutrophil-lymphocyte (NLR), monocyte-lymphocyte ratio (MLR), and the systemic immune inflammation index (SII), are well-recognized early biomarkers of systemic inflammation and immune dysregulation leading to organ failure (OF) in different clinical settings^1^ ^,^ ^2^. They have been shown to predict OF and mortality in patients admitted to the intensive care unit (ICU) in some^3^ ^-^ ^7^, but not all studies^8^ ^,^ ^9^. They have been associated with adverse outcomes, particularly in patients with community-acquired pneumonia^10^, sepsis and septic shock^11^ ^,^ ^12^, COVID-19 infection^13^, acute stroke^14^, coronary artery disease^15^ and trauma^16^ ^,^ ^17^.

Recent studies have also disclosed an association of leukocyte markers, particularly NLR, with adverse outcomes after surgery^18^ ^-^ ^27^, particularly cardiac^19^ ^-^ ^21^ and gastrointestinal (GI) surgery^22^ ^-^ ^27^, as well as after ICU admission due to acute pancreatitis^28^ ^,^ ^29^, small bowel obstruction^30^, and decompensated cirrhosis either with or without acute on chronic liver failure (ACLF)^31^ ^-^ ^33^, but few studies were designed to assess the ability of those biomarkers in the prediction of OF and survival of GI disorders leading to ICU admission.

The purpose of the present study was to evaluate the association of NLR, MLR, and SII, calculated at baseline just after ICU admission, with the requirement for organ support and mortality in critically ill patients admitted to a specialized ICU dedicated to the management of clinical and surgical patients with GI disorders. 

## METHODS

### Study population

This is a retrospective cohort study using electronic medical record data from all patients admitted to a specific gastrointestinal ICU, from January 2016 to December 2023. Participants were recruited using convenience sampling. All patients analyzed in this study had predefined causes of admission in the ICU, including postoperative care of major abdominal surgery, GI bleeding, acute abdomen, acute decompensated cirrhosis with or without acute-on-chronic liver failure (ACLF), acute cholangitis, severe liver injury with or without acute liver failure, pre-eclampsia, or eclampsia with liver enzymes abnormalities, and acute gastroenterocolitis. Subjects with other causes of admission unrelated to GI diseases were excluded from the study.

### Data collection

Data on demographics, causes of ICU admission, baseline leukocyte markers, sepsis diagnosis, and the need for circulatory, kidney, and respiratory failure support in the first 7 days after ICU admission, as well as ICU and hospital length of stay (LOS), were retrieved from electronic medical charts of all patients. The diagnosis of sepsis was considered arbitrary in patients admitted to the ICU with infections requiring antibiotics for more than three days. Circulatory failure was also arbitrarily defined as the need for vasopressors. On the contrary, kidney and respiratory failure were considered, respectively, as requirements for renal replacement therapy and mechanical ventilation. The main outcome was in-hospital mortality. Baseline white blood cell (WBC) counts and ratios, including NLR, LMR, and SII were calculated, as previously described^17^. Differential leucocyte counts were automatically produced for all white blood cell count estimation and reported to a minimum of three significant figures. Differential leucocyte counts were automatically produced using Sysmex® XT-4000i hematology analyzer equipped with a flow cytometer.

### Statistical analysis

Statistical analysis was performed using SPSS 25.0 software (IBM, USA). Categorical variables were reported as absolute numbers and percentages. Continuous variables were assessed using the Shapiro-Wilk test, and those with Gaussian distributions were reported as mean and standard deviation (SD), or as median and interquartile range (IQR) if skewed. Leukocyte markers were compared with different outcomes using the chi-square test or Fisher’s test for categorical variables or Student’s t-test or the Mann-Whitney U test for continuous variables when appropriate. Variables associated with OF and mortality in univariate analysis with a *P*<0.10 were entered in a multivariate logistic regression model using stepwise elimination. A *P* value <0.05 was considered significant.

### Ethical approval

The study was approved by the Ethics Committee in Research of the Portuguese Hospital of Salvador, Bahia, (CAAE 75898923.4.0000.5029) and conducted in accordance with the ethical standards of the Helsinki Declaration.

## RESULTS

**FIGURE 1:**
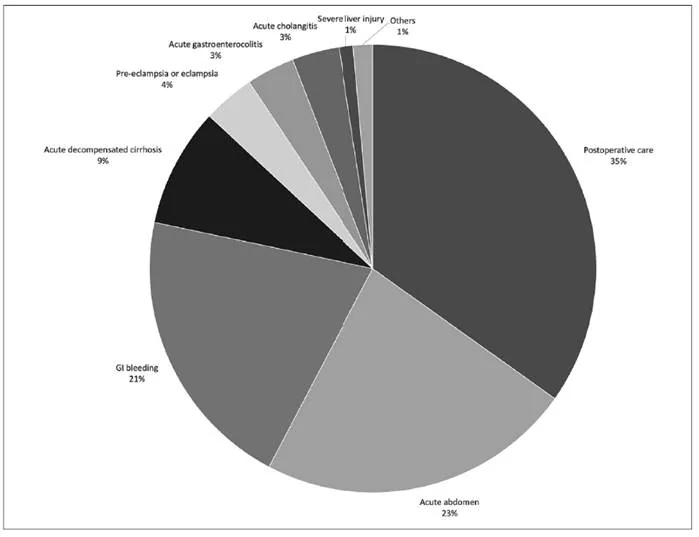
Main causes of GI admissions in the ICU.

7,673 patients were admitted to the ICU between January 2016 and December 2023. After exclusion of 970 subjects admitted for causes unrelated to GI diseases, 6703 patients (3593 females; mean age 67,4+18,3 years) were included in the study. The main causes of admission are depicted in Figure 1. The common causes of ICU admission were postoperative care (35%), acute abdomen (23%) and GI bleeding (21%) (Figure 1).

Demographics, clinical and laboratory features, and outcomes of those patients are summarized in Table 1. Briefly, the mean leukocyte, neutrophil, monocyte, and lymphocyte counts were, respectively 9900 (6480-14190), 7920 (4585-12048), 573 (317-878), and 931 (532-1443). Conversely, the mean NLR, MLR and SII were, respectively, 8.40 (4.32-16.96), 0.60 (0.34-1.00), and 1703 (764.76-3761.24). Roughly, 1/3 of the patients had sepsis either at baseline or in the first 7 days after ICU admission. Circulatory, respiratory, and kidney failure were observed in 14,8%, 5,55 and 1,8% of the patients, respectively. The median LOS in the ICU and in the hospital was 3,5 (1,8-6,5) and 8,0 (4,3-16,0) days. Eight hundred eighty-two (13,2%) patients died during hospitalization. 

**TABLE 1:** Clinical and Laboratory Features of Patients (n=6703).

Demographics	
Female sex	3593 (53,6%)
Age (years)	67,4±18,3
Leukocyte biomarkers	
- Leukocytes	9900 (6480-14190)
- Neutrophils	7920 (4585-12048)
- Monocytes	573 (317-878)
- Lymphocytes	931 (532-1443)
- Platelets	209 (149-280)
- NLR	8,40 (4,32-16,96)
- MLR	0,60 (0,34-1,00)
- SII	1703 (764,76-3761,24)
**Outcomes**	
Sepsis	1809 (27%)
Circulatory failure	989 (14,8%)
Respiratory failure	368 (5,5%)
Kidney failure	122 (1,8%)
ICU LOS (days)	3,5 (1,8-6,5)
Hospital LOS (days)	8,0 (4,3-16,0)
Mortality	882 (13,2%)

NLR: neutrophil-lymphocyte ratio. MLR: lymphocyte-monocyte ratio. SII: systemic immune-inflammation index (SII). LOS: length of stay.

As expected, patients with sepsis had significantly higher leukocyte (10.230 (100-59.500) vs 9850 (120-127.640) leukocytes in subjects without sepsis, *P*=0,01) and neutrophil (8463 (0,0-56.525) vs 7853 (0,0-122.534) neutrophils in subjects without sepsis, *P*<0,0001) counts and lower lymphocyte (787 (0,0-7179) vs 970 (0,0-4911) lymphocytes in subjects without sepsis, *P*<0,0001) and platelet (205 (10-1391) vs 211 (4-1653) platelets in subjects without sepsis, *P*=0,001) counts, when compared to their counterparts without sepsis. Likewise, higher NLR (10,37 (0,03-134) vs 7,93 (0,09-194,6) in those subjects without sepsis, *P*<0.0001), MLR (0,69 (0,01-18) vs (0,57 (0,01-24,5) in those subjects without sepsis, *P*<0.0001) and SII (2013,8 (1,51-63290) vs 1630 (1,42-82337) in those subjects without sepsis, *P*<0.0001) were observed in those subjects with sepsis, but on univariate and multivariate only NLR and MLR were associated with sepsis occurrence (Table 2).

**TABLE 2:** Univariate and multivariate analysis of leukocyte markers according to the occurrence of sepsis, organ dysfunction and mortality.

	Univariate Anaylsis			Multivariate Anaylsis		
	OR	95%CI	*P* **value**	OR	95%CI	*P* **value**
Sepsis						
NLR	1.012	1.009-1.014	<0.0001	1.007	1.003-1.010	<0.0001
MLR	1.171	1.116-1.228	<0.001	1.066	1.008-1.128	0.025
Circulatory failure						
NLR	1.018	1.016-1.021	<0.0001	1.017	1.013-1.021	<0.0001
MLR	1.181	1.122-1.242	<0.0001			
Respiratory failure						
NLR	1.0011	1.007-1.016	<0.0001	1.007	1.001-1.012	0.018
MLR	1.143	1.074-1.216	<0.0001			
Kidney failure						
NLR	1.008	1.001-1.016	0.036			
Mortality						
NLR	1.018	1.015-1.021	<0.0001	1.016	1.012-1.019	<0.0001
MLR	1.210	1.149-1.276	<0.0001			

Analysis of leukocyte markers according to the occurrence of OF and mortality revealed that leukocyte and neutrophil counts were significantly higher in subjects with circulatory and respiratory, but not in those with kidney failure. Lymphocytes were significantly depleted in patients with any OF, whereas platelets were significantly lower in those patients with circulatory and respiratory failure and monocytes just in those with kidney failure. NLR and SII were also significantly higher in patients with cardiac, respiratory and kidney failure. MLR, in contrast, was found to be increased only in subjects with CF (Table 3). On univariate analysis, both NLR and MLR were associated with CF and RF, but only NLR was independently linked to CF (OR: 1.017; 95%CI: 1.013-1.021; *P*<0.0001) and RF (OR: 1.007; 95%CI: 1.001-1.012; *P*<0.0001) after multivariate analysis. On the contrary, NLR was associated with a greater risk for KF on univariate but not on multivariate analysis (Table 2). 

**TABLE 3:** Leukocyte markers in subjects admitted to the ICU according to the occurrence of organ failure.

Leukocyte Markers	Circulatory Failure			Respiratory failure			Kidney failure		
	Yes	No	*P*	Yes	No	*P*	Yes	No	*P*
Leukocytes	12090 (120-127640)	9625 (100-114510)	0,0001	12020 (120-127640)	9870 (100-114510)	0,0001	9225 (1700-60710)	9900 (100-127640)	0,742
Neutrophils	10037 (0,0-122534)	7600,50 (0,0-101914)	0,0001	9917 (0,0-122534)	7875 (0,0-101914)	0,0001	7805 (1051-47354)	7924 (0,0-122534)	0,808
Lymphocytes	767 (0,0-5711)	960 (0,0-49111)	0,0001	850,5 (0,0-4565)	931 (0,0-49111)	0,002	720 (108-4023)	936 (0,0-49111)	0,001
Monocytes	571 (0,0-4933)	576 (0,0-20879)	0,665	561,5 (0,0-5958)	577 (0,0-20879)	0,757	430,5 (25-2880)	576 (0,0-20879)	0,021
Platelets	202 (15-1391)	210 (4-1653)	0,034	210,5 (15-911)	210 (4-1653)	0,389	196,5 (41-546)	209 (4-1653)	0,848
NLR	12,53 (0,03-134,01)	7,82 (0,08-194,65)	0,0001	11,82 (0,17-104,96)	8,37 (0,03-194,65)	0,000	10,91 (1,52-97,09)	8,34 (0,03-194,65)	0,005
MLR	0,74 (0,01-20,99)	0,58 (0,01-24,51)	0,0001	0,65 (0,01-24,51)	0,60 (0,01-19,66)	0,179	0,59 (0,04-5,66)	0,60 (0,01-24,51)	0,719
SII	2451,50 (1,51-63290,50)	1580,88 (1,42-82337,28)	0,0001	2213,44 (8,80-54897)	1701,58 (1,42-82337,28)	0,000	1980,98 (213,12-46118,98)	1694,87 (1,42-82337,28)	0,012

Mortality, on the other hand, was more frequently observed in subjects with higher leukocyte and neutrophil counts and lower lymphocyte count and in patients with higher NLR, MLR and SII (Table 4). On univariate analysis, NLR and MLR were associated with a higher risk of in-hospital death, but only NLR was independently linked to mortality (OR: 1.016; 95%CI: 1.012-1.019; *P*<0.0001) (Table 2).

**TABLE 4:** Leukocyte markers in patients admitted to the ICU according to the occurrence of mortality.

	Mortality		*P*
	Yes	No	
Leukocytes	11125 (100-91220)	9740 (120-127640)	0,0001
Neutrophils	9085 (0,0-83922)	7716 (0,0-122534)	0,0001
Lymphocytes	749,5 (0,0-6988)	960 (0,0-49111)	0,0001
Monocytes	543,5 (0,0-12201,2)	576 (0,0-20879)	0,147
Platelets	196,5 (15-1391)	210 (4-1653)	0,082
NLR	12,28 (0,03-108,75)	7,90 (0,08-194,65)	0,0001
MLR	0,75 (0,01-20,99)	0,58 (0,01-24,51)	0,0001
SII	2373,67 (1,42-78775)	1607,65 (8,80-82337,28)	0,0001

## DISCUSSION

Our data have shown that leukocyte markers, particularly NLR and MLR, were associated with ongoing sepsis, OF, and mortality in critically ill patients with GI disorders, but only NLR was independently associated with CF, RF, and mortality. To our knowledge, this study is the largest cohort to date evaluating the impact of leukocyte indices in clinical and surgical patients admitted to a GI ICU. However, it should be mentioned that several other studies have addressed the prognostic role of those biomarkers, notably NLR, in the outcome of patients placed in general ICUs after major GI surgery^22^ ^,^ ^24^ ^,^ ^25^, GI bleeding^34^, acute abdomen^28^ ^-^ ^30^, decompensated cirrhosis^31^ ^-^ ^33^.

Several leukocyte markers have been linked to increased morbidity and mortality after GI surgery^22^ ^-^ ^25^. The most extensively studied was NLR. Josse et al.^22^ have investigated the influence of NLR on the occurrence of postoperative (PO) complications after colorectal surgery. The authors reported an association between NLR, particularly values >2.3, and complications following colorectal resections. Bi et al.^24^, on the other hand, reported that higher NLR values were associated with postoperative acute kidney injury (AKI) in patients admitted to the ICU after gastrointestinal and hepatobiliary surgery. NLR was additionally associated with worse survival after pancreatic cancer resection in a meta-analysis including 8 studies involving 1519 patients^25^. Other studies, which have used preoperative NLR before ICU have also been shown to have prognostic accuracy to predict adverse outcomes after bariatric surgery and emergency laparotomy^23^ ^,^ ^26^.

Investigators from the North American Consortium for the Study of End-stage Liver Disease (NACSELD) reported an independent association of NLR with 90-day mortality in acutely decompensated patients with cirrhosis. Bernsmeier et al. have also found that NLR was independently associated with 90-day mortality in ACLF patients, as defined by CLIF criteria, irrespective of CLIF-SOFA and MELD values. The same results were also reproduced in another study from Eastern Europe. Other leukocyte biomarkers were not evaluated in the studies.

NLR was further associated with adverse outcomes in patients with acute pancreatitis^27^ ^-^ ^29^, small bowel obstruction^30^ and GI bleeding^31^. Most of those reports have evaluated NLR solely as a prognostic marker for OF and mortality. In the present study, other leukocyte markers, including MLR and SII, were also evaluated, but only NLR was independently associated with CF, RF, and mortality. Higher NLR values may reflect neutrophilia and/or lymphopenia, well-known indicators of ongoing inflammation and immune paralysis in patients with and without sepsis^2^.

It is important to highlight that our study has several limitations due to its retrospective design and lack of comparison with other commonly used prognostic markers in the ICU, like APACHE II, SAPS, and the Charlson comorbidity index scores, but it underscores in a large sample size the prognostic role of those biomarkers in the ICU.

## CONCLUSION

In summary, this study demonstrates that baseline leukocyte indices, particularly NLR, are reliable prognostic markers of OF and mortality in critically ill patients. They can be automatically calculated using a complete blood count obtained at ICU admission, providing an inexpensive, readily available prognostic tool for use at least in patients with GI disorders admitted to the ICU.

## Data Availability

Data-available-upon-request

## References

[1] Song M, Graubard BI, Rabkin CS, Engels EA (2021). Neutrophil-to-lymphocyte ratio and mortality in the United States general population. Sci Rep.

[2] Buonacera A, Stancanelli B, Colaci M, Malatino L (2022). Neutrophil to Lymphocyte Ratio: An Emerging Marker of the Relationships between the Immune System and Diseases. Int J Mol Sci.

[3] Chen JJ, Kuo G, Fan PC, Lee TH, Yen CL, Lee CC (2022). Neutrophil-to-lymphocyte ratio is a marker for acute kidney injury progression and mortality in critically ill populations: a population-based, multi-institutional study. J Nephrol.

[4] Nashwan AJ, Othman MI, Ananthegowda DC, Singh K, Ibraheem A, Janardhanan JP (2025). Neutrophil-to-Lymphocyte Ratio Predicts Dialysis Timing & Prognosis in Critically Ill Patients. Health Sci Rep.

[5] Huang Z, Fu Z, Huang W, Huang K (2020). Prognostic value of neutrophil-to-lymphocyte ratio in sepsis: A meta-analysis. Am J Emerg Med.

[6] Ham SY, Yoon HJ, Nam SB, Yun BH, Eum D, Shin CS (2020). Prognostic value of neutrophil/lymphocyte ratio and mean platelet volume/platelet ratio for 1-year mortality in critically ill patients. Sci Rep.

[7] Wu X, Luo Q, Su Z, Li Y, Wang H, Liu Q (2021). Neutrophil-to-lymphocyte ratio as a predictor of mortality in intensive care unit patients: a retrospective analysis of the Medical Information Mart for Intensive Care III Database. BMJ Open.

[8] Zhou T, Zheng N, Li X, Zhu D, Han Y (2021). Prognostic value of neutrophil- lymphocyte count ratio (NLCR) among adult ICU patients in comparison to APACHE II score and conventional inflammatory markers: a multi center retrospective cohort study. BMC Emerg Med.

[9] Çakin Ö, Karaveli A, Yüce Aktepe M, Gümüş A, Yildirim ÖE (2024). Comparison of Inflammatory Marker Scoring Systems and Conventional Inflammatory Markers in Patients over 65 Years of Age Admitted to the Intensive Care Unit: A Multicenter, Retrospective, Cohort Study. J Clin Med.

[10] Kuikel S, Pathak N, Poudel S, Thapa S, Bhattarai SL, Chaudhary G (2022). Neutrophil-lymphocyte ratio as a predictor of adverse outcome in patients with community-acquired pneumonia: A systematic review. Health Sci Rep.

[11] Huang Z, Fu Z, Huang W, Huang K (2020). Prognostic value of neutrophil-to-lymphocyte ratio in sepsis: A meta-analysis. Am J Emerg Med.

[12] Xia Y, Xu H, Xie J, Niu H, Cai X (2024). Prognostic value of neutrophil-to-monocyte/lymphocyte ratio for 28-day mortality in ICU sepsis patients: a retrospective cohort study. Front Med.

[13] Farias JP, Silva PPCE, Codes L, Vinhaes D, Amorim AP (2022). Leukocyte ratios are useful early predictors for adverse outcomes of COVID-19 infection. Rev Inst Med Trop Sao Paulo.

[14] Li W, Hou M, Ding Z, Liu X, Shao Y, Li X (2021). Prognostic Value of Neutrophil-to-Lymphocyte Ratio in Stroke: A Systematic Review and Meta-Analysis. Front. Neurol.

[15] Li Y, Chen D, Fan Y, Zhu Q, Deng H, Chai X (2025). Association between neutrophil to lymphocyte ratio and all-cause mortality in critical patients with coronary artery disease - a study based on the MIMIC-IV database. Front Cardiovasc Med.

[16] Arslan K, Sahin AS (2024). Prognostic value of systemic immune-inflammation index, neutrophil-lymphocyte ratio, and thrombocyte-lymphocyte ratio in critically ill patients with moderate to severe traumatic brain injury. Medicine.

[17] Xu J, Li S, Lui KY, Song X, Hu X, Cao L (2022). The neutrophil-to-lymphocyte ratio: A potential predictor of poor prognosis in adult patients with trauma and traumatic brain injury. Front Surg.

[18] Zhu Y, Bi Y, Liu B, Zhu T (2023). Assessment of prognostic value of preoperative neutrophil-to-lymphocyte ratio for postoperative mortality and morbidity. Front Med.

[19] Haran C, Gimpel D, Clark H, McCormack DJ (2021). Preoperative Neutrophil and Lymphocyte Ratio as a Predictor of Mortality and Morbidity After Cardiac Surgery. Heart Lung Circ.

[20] Wang Q, Li J, Wang X (2021). The neutrophil-lymphocyte ratio is associated with postoperative mortality of cardiac surgery. J Thorac Dis.

[21] Zhu Y, Peng W, Zhen S, Jiang X (2021). Postoperative Neutrophil-to-Lymphocyte Ratio Is Associated with Mortality in Adult Patients After Cardiopulmonary Bypass Surgery: A Cohort Study. Med Sci Monit.

[22] Josse JM, Cleghorn MC, Ramji KM, Jiang H, Elnahas A, Jackson TD (2016). The neutrophil/lymphocyte ratio predicts major perioperative complications in patients undergoing colorectal surgery. Color. Dis.

[23] Alsabani MH, Alenezi FK, Alotaibi BA, Alotaibi AA, Olayan LH, Aljurais SF (2024). Ratios of Neutrophils and Platelets to Lymphocytes as Predictors of Postoperative Intensive Care Unit Admission and Length of Stay in Bariatric Surgery Patients: A Retrospective Study. Medicina.

[24] Bi JB, Zhang J, Ren YF, Du ZQ, Wu Z, Lv Y (2020). Neutrophil-to-lymphocyte ratio predicts acute kidney injury occurrence after gastrointestinal and hepatobiliary surgery. World J Gastrointest Surg.

[25] Mowbray NG, Griffith D, Hammoda M, Shingler G, Kambal A, Al-Sarireh B (2018). A meta-analysis of the utility of the neutrophil-to-lymphocyte ratio in predicting survival after pancreatic cancer resection. HPB.

[26] Shrihari V, Baloorkar R, Kannur SS, Patil SS, Gharpure RD (2024). A Prospective Observational Study to Assess the Neutrophil-to-Lymphocyte Ratio (NLR) and C-reactive Protein-to-Albumin Ratio (CAR) in Predicting Morbidity and Mortality Among Patients Undergoing Emergency Abdominal Surgery. Cureus.

[27] Vincent A, CA S (2024). Predicting Severity of Acute Pancreatitis-Evaluation of Neutrophil-to-Lymphocyte Count Ratio as Emerging Biomarker: A Retrospective Analytical Study. Cureus.

[28] Mihoc T, Tarta C, Duta C, Lupusoru R, Dancu G, Oprescu-Macovei MA (2021). Monitoring Approach of Fatality Risk Factors for Patients with Severe Acute Pancreatitis Admitted to the Intensive Care Unit. A Retrospective, Monocentric Study.. Diagnostics.

[29] Halaseh SA, Kostalas M, Kopec C, Toubasi AA, Salem R (2022). Neutrophil-to-Lymphocyte Ratio as an Early Predictor of Complication and Mortality Outcomes in Individuals With Acute Pancreatitis at a UK District General Hospital: A Retrospective Analysis. Cureus.

[30] Yoon JB, Lee SH (2021). The neutrophil-to-lymphocyte ratio has feasible predictive value for hospital mortality in patients with small bowel obstruction in the emergency department. Am J Emerg Med.

[31] Bernsmeier C, Cavazza A, Fatourou EM, Theocharidou E, Akintimehin A, Baumgartner B (2020). Leucocyte ratios are biomarkers of mortality in patients with acute decompensation of cirrhosis and acute-on-chronic liver failure. Aliment Pharmacol Ther.

[32] Chiriac S, Stanciu C, Singeap AM, Sfarti CV, Cuciureanu T, Trifan A (2020). Prognostic value of neutrophil-to-lymphocyte ratio in cirrhotic patients with acute-on-chronic liver failure. Turk J Gastroenterol.

[33] Rice J, Dodge JL, Bambha KM, Bajaj JS, Reddy KR, Gralla J, Ganapathy D, Mitrani R, Reuter B, Palecki J, Acharya C, Shaw J, Burton JR, Biggins SW (2018). Neutrophil-to-Lymphocyte Ratio Associates Independently With Mortality in Hospitalized Patients With Cirrhosis. Clin Gastroenterol Hepatol.

[34] Chen X, Li X, Zhao G, Xu W (2024). Neutrophil-lymphocyte ratio predict outcome of upper gastrointestinal bleeding in emergency. Front Med.

